# Comparison of clinical characteristics and outcomes in primary neuroendocrine breast carcinoma versus invasive ductal carcinoma

**DOI:** 10.3389/fonc.2024.1291034

**Published:** 2024-05-10

**Authors:** Li Peng, Mingwei Ma, Dachun Zhao, Jialin Zhao, Qiang Sun, Feng Mao

**Affiliations:** ^1^ Department of Breast Surgery, Peking Union Medical College Hospital, Chinese Academy of Medical Science and Peking Union Medical College, Beijing, China; ^2^ Department of General Surgery, Peking Union Medical College Hospital, Chinese Academy of Medical Science and Peking Union Medical College, Beijing, China; ^3^ Department of Pathology, Peking Union Medical College Hospital, Chinese Academy of Medical Science and Peking Union Medical College, Beijing, China

**Keywords:** neuroendocrine breast carcinoma, invasive carcinoma of no special type, clinical characteristics, case-control study, prognosis

## Abstract

**Background:**

Neuroendocrine breast carcinoma (NECB) is a rare, special histologic type of breast cancer. There are some small sample studies on the clinical outcomes of NECB patients, which are worthy of further discussion.

**Methods:**

We conducted a retrospective case-control study of clinical characteristics and outcomes among patients with primary NECB versus invasive carcinoma of no special type (NST) between November 2004 and November 2017 in the Peking Union Medical College Hospital, Beijing. NST patients were strictly matched 1:4 during the same period based on the TNM stage. Statistical comparisons were performed to determine the differences in survival between NST and NECB patients and to identify clinical factors that correlate with prognosis.

**Results:**

A total of 121 participants affected by primary NECB were included in our analysis from November 2004 to November 2017. Elderly persons (>60 years of age) were more likely to have primary NECB than young persons (p=0.001). In addition, primary NECB patients had significantly higher odds of having tumors 2-5 cm (36.5%) and >5 cm (6.1%) in size than NST patients. Despite a significant difference in tumor size, the proportion of patients with lymph node metastases showed no difference between the two groups (p=0.021). In addition, the rate of patients with ER-negative tumors in the NECB group (4.2%) was significantly lower than that in the primary NST group (29.8%). Significant differences were noted in the PR-negative (13.3% versus 36.6%, P<0.001) and HER2-negative (90.5% versus 76.4%, P=0.001) expression statuses among these patients. Of 121 primary NECB patients, 11 (9.1%) experienced relapses during the follow-up period. We found that tumor size was an independent risk factor for relapse. For hormone receptors on tumor cells, ER-positive breast cancer patients had significantly lower odds of relapse than receptor-negative patients.

**Conclusions:**

Our data demonstrate no significant difference in mortality and relapse between the primary NECB and NST groups. The tumor size in the primary NECB group was significantly larger than that in the NST group. In addition, the absence of ER independently increased the relapse rate for breast carcinoma patients.

## Introduction

Breast cancer is the most frequently diagnosed malignancy and is a leading cause of cancer deaths in females worldwide. This form of cancer represents 12% of all new incident cancer cases and one-quarter of all cancers in women (1). In past decades, significant progress has been achieved in diagnosis and therapeutic strategies engaged in breast cancer management (2). Unfortunately, poor prognosis in patients affected by breast cancer remains of great concern among the population of underdeveloped areas due to diagnostic delay (3). More efforts are needed to achieve health equity to reduce the mortality associated with breast cancer in women.

According to tumor location, size, histology, and grade (4), significant diversity is noted in differentiation and proliferative activity across subgroups, mirroring its aggressiveness and prognosis (5). The most frequently diagnosed forms of breast cancer include invasive carcinoma of no special type, without tissue of origin(NST), constituting 80%–90% of all cases (5). In addition, NECB was first recognized in 1963 and is a rare special histologic type of breast cancer (6, 7). This special tumor differs in pathogenesis from others that have similar morphologic and phenotypic characteristics to digestive and pulmonary neuroendocrine tumors (7, 8). Therefore, NECB patients may have a different disease trajectory than those with other breast tumors, which triggers whether these patients represent a heterogeneous group of disease entities with different outcomes (9, 10). Unfortunately, its reported prevalence ranged from 0.1% to 15%, depending on the study series (7). Several studies have documented breast NECB studies with survival associations, but information on the clinical outcomes of patients with NECB is still lacking and needs further exploration (8, 11). More insights into NECB are required to guide treatment decision-making and optimize the design of clinical trials.

To address this concern, we conducted a retrospective study of clinical characteristics and outcomes among patients with primary NECB versus NST in the Peking Union Medical College Hospital, Beijing. Our objectives were to determine the differences in survival between these two groups and to identify clinical factors that correlate with prognosis.

## Methods

### Study design

**Figure 1:**
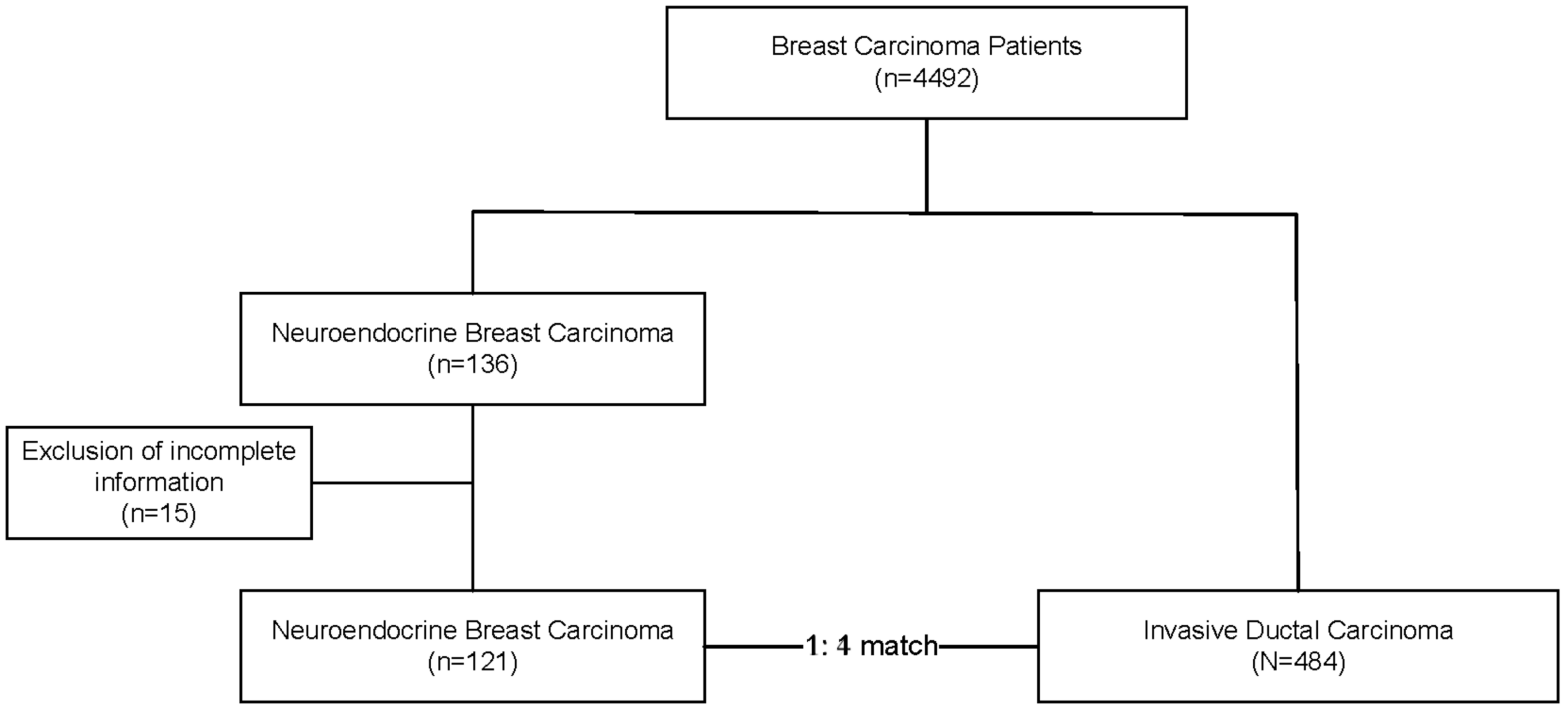
The flowchart of our study.

A series of 131 primary NECB cases were retrieved from the electronic records of the Peking Union Medical College Hospital between November 2004 and November 2017. Two pathologists reviewed representative histological slides to confirm the diagnosis. Inclusion criteria included (1) patients who had a first cancer diagnosis of primary NECB, (2) metastases from the GI tract NECBs were excluded, and (3) patients who completed the follow-up in our hospital. The primary NECB tumors were diagnosed following the guidelines of the World Health Organization (12). They were characterized by densely packed hyperchromatic cells with scant cytoplasm, streaming, and crush artifacts. Either chromogranin A (CgA) or synaptophysin (Syn) expression was observed in up to 50% of tumor cells. In addition, the hormone estrogen receptor (ER) and progesterone receptor (PR) were highly expressed, but human epidermal growth factor 2 (HER2) was negative according to ISH results. To assess the potential impact on the survival of primary NECB patients, NST patients were strictly matched 1:4 during the same period based on the TNM stage. One hundred thirty-one cases were retrieved from the medical records and re-evaluated. Ten cases were excluded after the re-evaluation, and 121 cases were finally included in this study. The flowchart of the study was shown in **Figure 1**. This study was approved by the Ethics Committee of Peking Union Medical College Hospital. The institutional review board approved a waiver of patient informed consent because of the anonymization of patient data and presentation of no more than minimal risk of harm to patient subjects.

### Data collection

**Figure 2:**
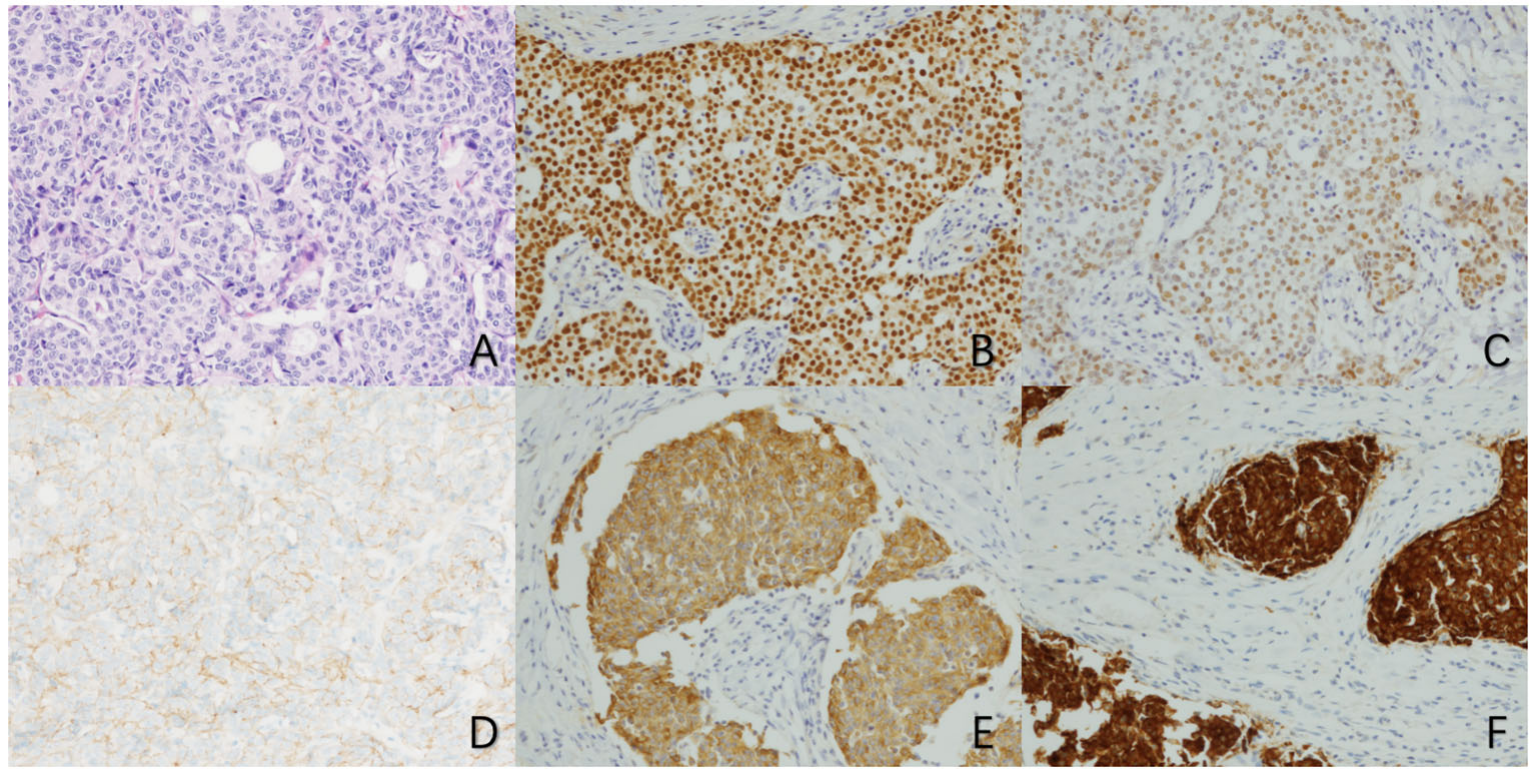
Microscopic manifestations of primary neuroendocrine carcinoma of the breast (×100) **(A)** HE staining shows a large number of cancer cells with tubular and trabecular structures. **(B)** Immunohistochemical staining for ER showed strong positivity in tumor cells. **(C)** Immunohistochemical staining for PR showed moderate positivity in tumor cells. **(D)** Immunohistochemical staining for HER2 showed no significant staining of tumor cells. **(E)** Immunohistochemical staining for CgA showed diffuse positive staining in tumor cells. **(F)** Immunohistochemical staining for Syn showed diffuse positive staining in tumor cells).

The electronic medical record system documented morbidity, treatment, and care over time. Demographic and clinical variables were collected from electronic medical records to compare the primary NECB and NST groups, including sex, age, place of residence, and comorbidities. In addition, the histological findings were also retrieved from electronic medical records, including morphology and expression levels of neuroendocrine and hormone markers. A cutoff of 1% expression or greater was used to define ER and PR positivity (13). For neuroendocrine markers, CgA, Syn, and neuron-specific enolase (NSE) were considered positive if at least 50% of tumor cells demonstrated expression of each marker (14). According to the previous guidelines, HER2 was scored using both percent positive and intensity, and only tumors expressing HER2 in ≥30% of cells at 3+ intensity were considered positive. The Ki-67 expression was defined as the percentage of tumor cells with nuclear Ki-67 staining. Based on the 2013 St. Gallen consensus standard (15), the patients affected by breast cancer were divided into four subtypes. In addition, internationally recognized TNM staging systems were used to classify malignant tumors. The immunohistochemical staining of tumor cells is shown in **Figure 2**.

The tumor subtypes were classified as Luminal A (ER and PR positive, HER2 negative, ‘low’ Ki-67, and a ‘low’ recurrence risk based on multi-gene-expression assay results if available), Luminal B (‘Luminal B-like (HER2 negative)’: ER positive, HER2 negative, and at least one of the following: ‘high’ Ki-67, ‘negative or low’ PR, or ‘high’ recurrence risk based on multi-gene-expression assay if available. ‘Luminal B-like (HER2 positive)’: ER positive, HER2 over-expressed or amplified with any Ki-67, and any PR), HER2+ (Hormone receptor-negative and HER2-positive), and triple negative (TN) (Negative ER, PR, and HER2) according to St. Gallen’s Guide in 2013 (16).

### Statistical analysis

The anonymization of patient data was conducted prior to analysis. SPSS version 20.0 (IBM Corp, Armonk, NY) was used for the statistical calculations. Numbers and proportions of cases and various demographic and clinical characteristics were tabulated. The mean and standard deviation (SD) are presented for normally distributed continuous variables, while the median and interquartile range (IQR) present nonnormally distributed continuous variables. As requested, statistical comparisons were performed using the Student’s *t-test*, Chi-square test, and Fisher’s exact test. A survival curve was constructed with the Kaplan–Meier method. Two-sided tests with P<0.05 were considered statistically significant.

## Results

### Patient characteristics and follow-up results

We reviewed the consecutive pathology findings for 4492 women with breast cancers diagnosed between November 2004 to November 2017 in our hospital. A total of 121 patients were diagnosed with primary NECB based on histopathology results. The clinical characteristics of the included patients are shown in **Table 1**.

**Table 1:** Patient characteristics of patients undergoing primary NECB versus NST.

Patient characteristics	NECB		NST		Total		t/χ^2^	*P*
	No.	%	No.	%	No.	%		
**Median age- year**	54.0(41.0-65.0)		51.0(44.0-58.0)				-0.148	0.139
**Age**							13.458	0.001
-39 years	22	18.2	65	13.4%	87	14.4		
40-59 years	56	46.3	311	64.3	367	60.7		
60- years	43	35.5	108	22.3	151	25.0		
**Relapse**							0.700	0.403
No	110	90.9	427	88.2	531	88.8		
Yes	11	9.1	57	12.6	74	11.2		
**Tumor size**							7.755	0.021
≤2cm	66	57.4	331	68.4	397	66.3		
2cm-5cm	42	36.5	142	29.3	184	30.7		
>5cm	7	6.1	11	2.3	18	3.0		
**No. dissected lymph nodes**	18(5.74-23.25)		19.0(15.0-24.0)				-2.929	0.003
**No. lymph node metastases**							0.712	0.870
0	74	61.2	279	57.6	353	58.3		
1~3	26	21.5	108	22.3	134	22.1		
4~9	11	9.1	55	11.4	66	10.9		
≥10	10	8.3	42	8.7	52	8.6		
**ER**							33.873	<0.001
Negative	5	4.2	144	29.8	149	24.7		
Positive	115	95.8	340	70.2	455	75.3		
**PR**							23.879	<0.001
Negative	16	13.3	177	36.6	193	32.0		
Positive	104	86.7	307	63.4	411	68.0		
**HER2**							11.241	0.001
Negative	105	90.5	363	76.4	468	79.2		
Positive	11	9.5	112	23.6	123	20.8		
**Ki-67 expression**							0.652	0.419
≤14%	37	31.4	132	27.6	169	28.4		
>14%	81	68.6	346	72.4	427	71.6		
**Tumor Grade**							6.809	0.078
I	18	14.9	59	12.2	77	12.7		
II	61	50.4	246	50.8	307	50.7		
III	31	25.6	159	32.9	190	31.4		
Unknown	11	9.1	20	4.1	31	5.1		
LVSI							181.835	0
Negative	22	18.2	395	81.6	417	68.9		
Positive	99	81.8	89	18.4	188	31.1		
**Associated intraductal carcinoma component**							0.399	0.528
Yes	20	16.5	69	14.3	89	14.7		
No	101	83.5	415	85.7	516	85.3		
**Therapy**							12.705	0.013
Surgery	121	100	484	100	605	100.0		
Chemotherapy	65	53.7	323	66.7	388	64.1		
Radiotherapy	50	41.3	151	31.2	201	33.2		
Endocrine therapy	108	89.3	358	74.0	466	77.0		
Anti-HER2 therapy	11	9.1	85	17.6	96	15.9		
**subtype**							26.982	<0.001
Luminal A	32	26.4	102	21.1	134	22.1		
Luminal B	78	64.5	254	52.5	332	54.9		
HER2 subtype	0	0	51	10.5	51	8.4		
TN	4	3.3	66	13.6	70	11.6		
Unknown	5	4.1	11	2.3	16	2.6		

ER, Estrogen receptor; PR, progesterone receptor; HER2, human epidermal growth factor 2.

Of these, 78 patients had stage I or II disease, 22 had stage III or IV disease, and 21 patients could not be staged. 67 patients (55.4%) received mastectomy + axillary lymph node dissection and 16 patients (13.2%) received mastectomy + sentinel lymph node biopsy. 7 patients (5.8%) received lumpectomy + axillary lymph node dissection and 15 patients (12.4%) received lumpectomy + sentinel lymph node biopsy. 16 patients (13.2%) received lumpectomy without axillary stage due to the old age.

More than half of the patients(66/121, 54.5%) received chemotherapy to treat primary breast cancer. Most patients received adjuvant chemotherapy; the interval between surgery and adjuvant chemotherapy was 2-4 weeks. Two patients received neoadjuvant chemotherapy. HER2-negative patients received doxorubicin/epirubicin + cyclophosphamide, docetaxel + cyclophosphamide, paclitaxel/docetaxel + doxorubicin, or docetaxel + doxorubicin/epirubicin + cyclophosphamide, and HER2-positive patients received docetaxel + cyclophosphamide + trastuzumab and doxorubicin + cyclophosphamide followed by docetaxel + trastuzumab or trastuzumab+pertuzumab. Regimens included doxorubicin/epirubicin 50/75 mg/m2, cyclophosphamide 500 mg/m2, paclitaxel/docetaxel 175/75 mg/m2 every 3 weeks, trastuzumab 8 mg/kg IV on day 1 followed by 6 mg/kg every 3 weeks, and pertuzumab 840 mg for the first dose followed by 420 mg every 3 weeks. The duration of anti HER2 therapy was one year. The above methods and doses of radiotherapy and chemotherapy are also applicable to IDC group.

Fifty patients received adjuvant radiotherapy to treat primary breast cancer. For breast conserving therapy, whole-breast irradiation was delivered via opposed tangential fields using a regimen of 50Gy in 2Gy daily fractions with 6Mv-X rays from a linear accelerator. Invasive disease was treated with a boost of 10Gy in 5 fractions to the tumor bed and 1-2 cm margins. Regional nodal irradiation included the lower part of the ipsilateral axillary LN in all cases and the upper part of the ipsilateral axillary LN when there were metastases to the LNs in the axilla. Postmastectomy, 45-50Gy at 2Gy/fx with 6Mv-X rays was delivered from a linear accelerator to a target volume that included the chest wall and supraclavicular fossa. High risk patients were treated with an electron boost to bring the scar dose to 60-66Gy.

The majority of patients (109/121, 90.1%) received endocrine therapy to treat primary breast cancer, including tamoxifen, 10 mg twice a day or 20 mg once a day; letrozole, 2.5 mg once a day; anastrozole, 1 mg once a day; exemestane, 25 mg once a day; or a goserelin acetate 3.6mg depot implanted subcutaneously very 4 weeks. The duration of adjuvant endocrine therapy was 5 years for most patients, and 5-10 years for the patients with high risk of recurrence.

In the primary NECB group, the main types were luminal A and B, accounting for 26.4% and 64.5%, respectively. TN subtype accounted for 3.3%, and there was no Her2 subtype among these patients. In the NST group, the proportion of subtypes tended to be similar to that reported in the literature(luminal A 21.1%, luminal B 52.5%, Her2 10.5% and TN 13.6%). There were statistical differences in the subtypes between primary NECB and NST (P < 0.05) (17).

### Demographic and risk factor characteristics of primary NECB patients

One hundred twenty-one participants affected by primary NECB were included in our analysis from November 2004 to November 2017, while 484 NST patients were matched based on the TNM stage as a control group during the same period. The detailed comparison of demographic and clinical characteristics is summarized in **Table 1**. The median ages of patients with primary NECB and NST were 54.0 (41.0-65.0) and 51.0 (44.0-58.0), respectively. The distribution of the two groups showed significant differences stratified into various age groups. We found that elderly persons (>60 years of age) were more likely to have primary NECB than young persons (p=0.001). In addition, primary NECB patients had significantly higher odds of having tumors 2-5 cm (36.5%) and >5 cm (6.1%) in size than NST patients. Despite a significant difference in tumor size, the proportion of patients with lymph node metastases showed no difference between the two groups (p=0.021). In addition, there was a significant difference in ER, PR, and HER2 expression between the primary NECB and NST groups. The rate of patients with ER-negative tumors in the primary NECB group (4.2%) was significantly lower than that in the NST group (29.8%)(p<0.001). Significant differences were noted in the PR-negative (13.3% versus 36.6%, P<0.001) and HER2-negative (90.5% versus 76.4%, P=0.001) expression statuses among these patients. We observed no significant difference in relapse between the two groups.

### Risk factors associated with relapse in primary NECB patients

Of 121 primary NECB patients, 10 (10.7%) experienced relapses during the follow-up period. We further analyzed the risk factors associated with relapse in primary NECB patients. As summarized in **Table 2**, tumor size was an independent risk factor for relapse. The relapse rate in tumors of 2-5 cm (17.4%) was significantly higher than that in tumors less than 2 cm (aOR: 2.206, 95% CI: 1.244-3.912). For hormone receptors on tumor cells, we found that ER-positive breast cancer patients had significantly lower odds of relapse than ER-negative patients (aOR 0.235, 95% CI 0.135-0.410). Similarly, patients with lymph node metastasis had a higher risk for relapse than those without (OR 2.371, 95% CI 1.325-4.244).

**Table 2:** Factors associated with relapse among primary NECB patients enrolled in this study.

Patient characteristics	Non-Relapse		Relapse		Crude OR (95% CI)	Adjusted OR (95% CI)
	NO.	%	NO.	%		
Age						
-39 years	74	13.8	13	19.1		
40-59 years	326	60.7	41	60.3	0.716 (0.365-1.403)	
60- years	137	25.5	14	20.6	0.582 (0.260-1.303)	
Tumor size						
≤2cm	368	68.8	29	45.3		
2cm-5cm	152	28.4	32	50.0	2.672 (1.562-4.570)	2.206 (1.244-3.912)
>5cm	15	2.8	3	4.7	2.538 (0.694-9.275)	1.191 (0.298-4.761)
Lymph node metastases						
No	327	60.9	26	38.2		
Yes	210	39.1	42	61.8	2.515 (1.497-4.226)	2.371 (1.325-4.244)
ER						
Negative	115	21.5	34	50.0		
Positive	421	78.5	34	50.0	0.273 (0.163-0.459)	0.235 (0.135-0.410)
PR						
Negative	156	29.1	37	54.4		
Positive	380	70.9	31	45.6	0.344 (0.206-0.574)	
HER2						
Negative	419	79.5	49	76.6		
Positive	108	20.5	15	23.4	1.188 (0.642-2.199)	
Ki-67 expression						
≤14%	159	30.1	10	14.9		
>14%	370	69.9	57	85.1	2.449 (1.22-4.919)	

## Discussion

NECB is a rare type of breast cancer, and many cases remain undiagnosed due to its rarity and heterogeneity (7). In this study, we described and analyzed clinical characteristics and prognosis in the largest number of primary NECB patients from China. Our data demonstrated no significant difference in mortality and relapse between the primary NECB and NST groups. Consistent with our observation, several previous studies confirmed that patients afflicted with primary NECB had similar prognoses and clinical presentations compared with other breast carcinomas (6, 18). However, conflicting results were noted in a population-based study from the Surveillance, Epidemiology, and End Results (SEER) database, which indicated that primary NECB was associated with worse long-term outcomes (19). In addition, a few studies have revealed that primary NECB is a nonaggressive breast carcinoma type with a better prognosis (20, 21). These contradictory results might be explained by the limited number of cases reported in each cohort and varying inclusion criteria from the WHO definitions for identifying primary NECB. In addition, routine physical examinations have been widely conducted in recent years in China and are helpful for the early identification of breast cancer patients. Thus, we speculate that diagnosing these breast carcinomas at an early stage may be another possible explanation for the comparative outcomes between the two groups. Consistent with our hypothesis, most of our primary NECB participants were classified as early T1-2 stage, which significantly contributed to the low relapse rate.

Despite no difference in prognosis between primary NECB and NST patients, we observed that the tumor size in the primary NECB group was significantly larger than that in the NST group. These findings are consistent with previous data that primary NECB presented with larger tumor size and high histological grade (22), which may reflect the faster intrinsic growth rate of primary NECB than NST. A previous study revealed that HER2- breast tumors often display higher proliferation rates than HER+ tumors (23). In our cohort, higher proportions of patients with ER-positive and HER2-negative breast cancer were noted in the primary NECB group than in the NST group. Thus, it is probable that the subtypes of HER2- breast cancer have an enhanced proliferation rate to achieve a larger tumor size at the time of diagnosis. Tumor size reflects the number of cancer cells and is also a predictor of outcome (23). Although we found no significant difference in relapse rate between the two groups due to the high potential for early diagnosis, our results imply a higher risk of poor clinical outcomes, including metastasis and short-term survival for patients with fast-growing primary NECB cancer.

A previous study by Wang and colleagues confirmed that primary NECB disease is more commonly diagnosed in older women in or above their sixth decade of life (19). Similar results were also reported in a retrospective analysis from China, demonstrating that primary NECB patients seemed to be older than the onset of the other tumor subtype (24). We also found that the proportion of patients with primary NECB aged >60 years was higher than that of the IDC group; however, approximately one-fifth of female patients were aged < 40 years. The diverse distribution of patients across age subgroups between primary NECB and NST indicates the difference in cancer pathogenesis. Specifically, hormone levels may play an essential role in the occurrence of primary NECB tumors, considering that this type of cell tends to express hormone receptors and lacks HER-2 (7, 25).

Due to the lack of an established standard treatment protocol, the treatment of primary NECB is consistent with that for other conventional types of invasive breast carcinomas. Based on our findings, routine therapies provided sufficient efficacy for the treatment of this rare breast cancer compared with other subtypes. The subsequent quantification of risk factors found that the absence of ER independently increased the relapse rate for breast carcinoma patients. Consistent with our findings, serial studies of breast carcinomas revealed that patients with ER-negative breast cancer had poor clinical outcomes (24, 26). More attention should be given to the follow-up of these patients at high risk of relapse. Previous studies on the prognostic significance of neuroendocrine differentiation in NECB have yielded contrary results due to different diagnostic criteria and the limited number of cases. In this study, we found that NECB tends to be a luminal-like type. There were only a small number of TN subtype patients and no patients with HER2 subtype in the queue. Despite all this, most recent studies have reported poorer clinical outcomes for NEBC compared with typical breast carcinomas (27, 28).

We also acknowledge several apparent limitations to this study. First, despite the enrollment of all primary NECB patients throughout the study period, the small number of patients associated with its low prevalence limits further analysis of risk factors for relapse in primary NECB patients. Second, the detailed treatment regimens were not considered in our analysis. Finally, our survival prognostic analysis of primary NECB patients was partially biased due to the low mortality of this study cohort. Despite these limitations, our study extends our knowledge about this rare breast cancer subtype.

In conclusion, our data demonstrate no significant difference in mortality and relapse between the primary NECB and NST groups. The tumor size in the primary NECB group was significantly larger than that in the NST group, and the primary NECB patients seemed to be older than the onset of the other tumor subtype. In addition, the absence of ER independently increased the relapse rate for breast carcinoma patients. Further clinical study is required to perform a prognostic analysis of the survival of primary NECB patients through long-term large-sample follow-up.

## Data availability statement

The original contributions presented in the study are included in the article/supplementary material. Further inquiries can be directed to the corresponding author.

## Ethics statement

The studies involving humans were approved by peking union medical college hospital ethics committee. The studies were conducted in accordance with the local legislation and institutional requirements. The participants provided their written informed consent to participate in this study.

## Author contributions

LP: Resources, Writing – original draft. MM: Software, Writing – original draft. DZ: Data curation, Resources, Writing – original draft. JZ: Data curation, Investigation, Methodology, Writing – original draft. QS: Supervision, Writing – review & editing. FM: Supervision, Writing – review & editing.
